# A preliminary study on the effects of red *Bonnemaisonia hamifera* seaweed on methane emissions from dairy cows

**DOI:** 10.3168/jdsc.2024-0670

**Published:** 2024-12-16

**Authors:** M. Thorsteinsson, A.A. Schönherz, S.J. Noel, Z. Cai, Z. Niu, A.L.F. Hellwing, P. Lund, M.R. Weisbjerg, M.O. Nielsen

**Affiliations:** 1 Department of Animal and Veterinary Sciences, AU Viborg, Research Centre Foulum, Aarhus University, DK-8830 Tjele, Denmark; 2 Center for Quantitative Genetics and Genomics, Aarhus University, DK-8000 C Aarhus, Denmark

## Abstract

The red seaweed *Bonnemaisonia hamifera* has shown potential as a CH_4_-mitigating feed additive in vitro, and hence, this pilot study aimed to investigate the potential of the seaweed when fed to dairy cows. The study was conducted as a 4 × 4 Latin square using 4 Danish Holstein cows with 4 periods of 7 d where the cows were placed in respiration chambers for the last 3 d. Milk yield and feed intake were recorded daily. The cows were fed a TMR without *B. hamifera* (control) or the same diet with the addition of, on a DM basis, 0.33% (low), 0.66% (medium), and 1% (high) *B. hamifera*. Increasing inclusion levels of *B. hamifera* linearly decreased CH_4_ emissions with up to a 13.3% reduction in daily CH_4_ production (g/d), 15.7% reduction in CH_4_ yield (g/kg DMI), and 16.4% reduction in CH_4_ intensity (g/kg ECM). In contrast, linear increases were observed in daily H_2_ production (g/d), H_2_ yield (g/kg DMI), and H_2_ intensity (g/kg ECM). The exact nature of the antimethanogenic compounds in *B. hamifera* is unknown; however, the concentration of the potentially harmful compound, bromoform, was remarkably lower in *B. hamifera* compared with reported concentrations in the known antimethanogenic red seaweed *Asparagopsis taxiformis.* In conclusion, *B. hamifera* showed CH_4_-mitigating properties in this preliminary study, but further in vivo studies are needed to fully evaluate the potential of the seaweed as an antimethanogenic feed additive.

## Highlights

• Methane yield was reduced by over 15% at 1% DM inclusion rate of *B. hamifera*.
• Hydrogen yield was increased by 325% at 1% DM inclusion rate of *B. hamifera*.
• The exact nature of the antimethanogenic compounds in *B. hamifera* is unknown.

## Graphical Abstract

**Figure:**
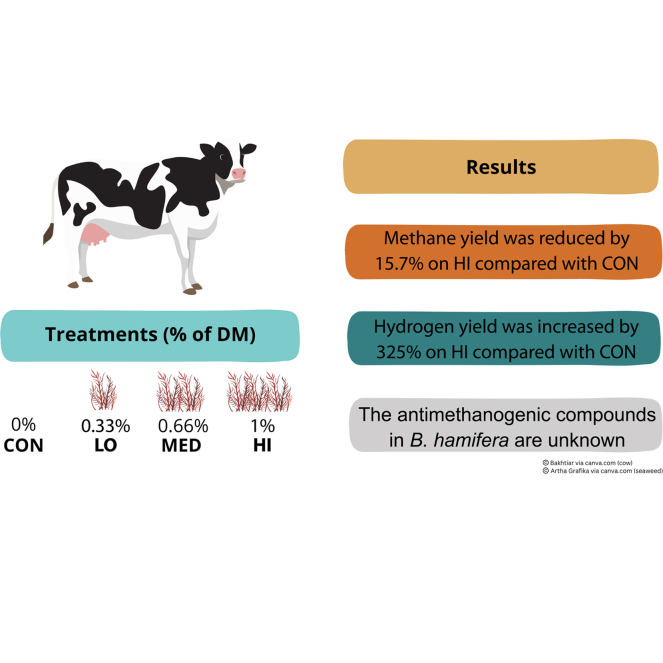
**Summary:** The red seaweed *Bonnemaisonia hamifera* has shown CH_4_-mitigating properties in several in vitro studies. This pilot study aimed to investigate the potential of the seaweed as an antimethanogenic feed additive when fed to dairy cows at a maximum inclusion rate of 1% on a dry matter (DM) basis. Increasing the level of *B. hamifera* resulted in linear decreases in daily CH_4_ production, yield, and intensity by 13% to 16%. Moreover, linear increases were observed in daily H_2_ emissions, yield, and intensity. Hence, larger and longer-term studies should be undertaken to fully investigate the potential of this seaweed because the concentration of the potentially harmful compound, bromoform, is remarkably lower than in the known antimethanogenic red seaweed *Asparagopsis* spp.

The CH_4_-mitigating properties of the red seaweed *Asparagopsis* spp. have been ascribed to their high concentrations of bromoform and other halomethanes (Machado et al., 2016), which have been known for decades to be efficient inhibitors of methanogenesis in ruminants (Lanigan, 1972). Hence, several recent in vitro and in vivo studies have demonstrated that the CH_4_-reducing effects are dependent on the dose of *Asparagopsis* spp., and thus the amount of bromoform (Kinley et al., 2016; Roque et al., 2019; Mihaila et al., 2022; Alvarez-Hess et al., 2024). However, in vivo studies with dairy cows have also reported reductions in feed intake and milk production (Roque et al., 2019; Stefenoni et al., 2021), abnormalities or damage to the rumen wall (Muizelaar et al., 2021), and transfers of bromoform metabolites into milk (Stefenoni et al., 2021; Krizsan et al., 2023) when fed *Asparagopsis* spp. Moreover, concerns regarding the contribution of *Asparagopsis* spp. cultivation to the depletion of the ozone layer due to the emission of bromoform have also been raised (Jia et al., 2022). Interestingly, Mihaila et al. (2022) reported that another red seaweed, *Bonnemaisonia hamifera*, reduced in vitro CH_4_ production by almost 96% at an inclusion level of 6% of OM, and in contrast to *Asparagopsis armata*, bromoform was not detectable in *B. hamifera* (Mihaila et al., 2022). The exact nature of the antimethanogenic bioactive compounds in *B. hamifera* is unknown, but Guinguina et al. (2023) speculated that 1,3,3-tetrabromo-2-heptanone and 1-iodo-3,3-dibromo-2-heptanone could be among the compounds as they are known to possess antibacterial and antimicrobial properties (Siuda et al., 1975; Nylund et al., 2008; Enge et al., 2012). To our knowledge, *B. hamifera* has never been evaluated in vivo as an antimethanogenic feed additive in ruminants, and hence, the objective of the current pilot study was to investigate the effects of increasing in practice applicable doses (0%–1% on a DM basis) of the red seaweed *B. hamifera* on the enteric CH_4_ emissions from dairy cows. It was hypothesized that increasing levels of *B. hamifera* would result in decreasing enteric CH_4_ emissions from dairy cows.

The experiment was conducted at Aarhus University, AU Viborg–Research Centre Foulum, Denmark, under a license from the Danish Animal Experiments Inspectorate. The experiment was planned under consideration of the ARRIVE Guidelines (Percie du Sert et al., 2020).

Four lactating Danish Holstein cows (2 first parity and 2 fifth parity) were included in a 4 × 4 Latin square design study. During the 4 periods, the cows received (1) basal control (**CON**; diet without any seaweed), (2) basal diet with 0.33% *B. hamifera* on a DM basis (**LO**), (3) basal diet with 0.66% *B. hamifera* on a DM basis (**MED**), or (4) basal diet with 1% *B. hamifera* on a DM basis (**HI**; Table 1). Each of the 4 experimental periods lasted 1 wk with 4 d of adaptation to the diet followed by 3 d of gas exchange measurements using respiration chambers. All cows were fed the CON diet in a pre-period (3 d) before the first period. At the initiation of the experiment, milk yield was 25.7 ± 12.0 kg/d (average ± SD), DIM was 209 ± 14.0 d, BCS was 3.0 ± 0.20 on a scale from 1 to 5, and BW was 685 ± 79.6 kg. The cows were housed in individual pens (400 × 450 cm) with slatted floor and a cubicle bed with a mattress and sawdust during adaptation periods. The cows had free access to water throughout the experiment.

**Table 1:** Dietary and chemical composition (means ± SD; % of DM unless otherwise stated) of the diet in the pre-period and a control diet without any seaweed and the same diet diluted with 3 different levels of the red seaweed *Bonnemaisonia hamifera*

Item	Treatment^1^				
	PRE	CON	LO	MED	HI
Dietary composition					
Spring barley	7.52	7.52	7.50	7.47	7.45
Rapeseed meals, 4% fat	17.5	17.5	17.5	17.4	17.4
Rapeseed cakes, 10.5% fat	4.18	4.18	4.16	4.15	4.14
Sugar beet pulp, rolled	7.10	7.10	7.08	7.06	7.03
Sugar beet molasses	4.18	4.18	4.16	4.15	4.14
Maize silage, silo 11–22	29.2	29.2	29.1	29.1	29.0
First cut grass/clover silage	14.6	14.6	14.6	14.5	14.5
Second regrowth grass/clover silage	14.2	14.2	14.2	14.1	14.1
Limestone	0.084	0.084	0.083	0.083	0.083
Sodium bicarbonate	0.627	0.627	0.625	0.623	0.620
Sodium chloride	0.125	0.125	0.125	0.125	0.124
Mineral premix^2^	0.418	0.418	0.416	0.415	0.414
Vitamins	0.146	0.146	0.146	0.145	0.145
*Bonnemaisonia hamifera*	0.00	0.00	0.33	0.66	1.00
Chemical composition					
DM (% of fresh feed)	43.2	44.5 ± 0.49	44.5 ± 0.52	44.7 ± 0.46	45.1 ± 0.53
Ash	7.23	7.35 ± 0.14	7.37 ± 0.05	7.43 ± 0.07	7.40 ± 0.05
CP	16.1	15.8 ± 0.59	16.0 ± 0.51	16.4 ± 0.33	15.9 ± 0.24
Crude fat	3.90	3.40 ± 0.18	3.48 ± 0.26	3.40 ± 0.26	3.43 ± 0.17
aNDFom^3^	32.4	32.4 ± 0.73	32.7 ± 0.25	32.5 ± 0.04	32.2 ± 0.62
Starch	14.8	14.3 ± 0.71	15.3 ± 1.04	15.0 ± 0.79	15.6 ± 1.26
NEL_20_^4^ (MJ/kg of DM)	6.46	6.46	—	—	—

1 PRE = diet in pre-period; CON = control diet; LO = 0.33% *B. hamifera*; MED = 0.66% *B. hamifera*; HI = 1% *B. hamifera* on a DM basis.

2 Vilofoss Komix Type 3, declared macromineral composition (g/kg DM): Ca = 147, Mg = 141, Na = 116, S = 1. Added vitamins and microminerals (per kg DM): vitamin A = 600,000.10 IU, vitamin D_3_ = 190,000.10 IU, vitamin E = 4,000 IU, Mn = 4,000 mg, Cu = 1,500 mg, Zn = 4,500 mg, Co = 25 mg, Se = 50 mg in combination with Vilofoss Suplex ADE, analyzed or declared macromineral composition (g/kg DM): Ca = 139, Mg = 91, Na = 95. Added vitamins and microminerals (per kg DM): vitamin A = 900,000 IU, vitamin D_3_ = 200,000 IU, vitamin E = 2,000 IU, Se = 50 mg.

3 aNDFom = NDF determined on an OM basis.

4 Net energy for lactation at 20 kg DMI/d, calculated according to NorFor (Volden, 2011).

Total mixed rations were prepared once a day and fed to the cows on an ad libitum basis with approximately 40% of the daily ration at 0615 h and 60% at 1600 h. The composition of TMR is shown in Table 1. Rations were formulated according to the Nordic Feed Evaluation System (Norfor; Volden, 2011) with an expected milk yield of 10,500 kg ECM per year. The roughage:concentrate ratio (on a DM basis) was 58:42 for all diets. Net energy for lactation in CON was calculated according to NorFor (Volden, 2011). Contents of crude fat, protein, starch, ash, and NDF on an OM basis (aNDFom) were determined as described by Thorsteinsson et al. (2023).

*Bonnemaisonia hamifera* was continuously cultivated in a land-based system by Maripure ApS (Aalborg, Denmark) throughout the experiment. After harvest, the biomass was freeze-dried and ground to a particle size of 2 to 4 mm and delivered in 4 different batches. Bromoform was analyzed in seaweed biomass using SIM mode on a GC-MS with a detection level of 1 µg of bromoform/g of DM as described by Nørskov et al. (2021). Across biomass batches, contents of ash were 182.4 ± 10.5 g/kg DM and bromoform 0.101 ± 0.027 mg/g DM.

Allocated feed and refusals were weighed daily throughout the experiment to determine feed intake, whereas DM in feed and refusals was measured from d 5 to 7 in each period by drying at 60°C for 48 h (Åkerlind et al., 2011). The DMI was calculated as the amount of DM in refusals subtracted from DM offered. The cows were milked twice daily at 0515 and 1615 h. Milk yield was recorded by continuous flowmeters at every milking throughout the experiment, while milk was analyzed for fat, protein, lactose monohydrate, and urea on d 5 to 7 in each period using mid-infrared reflection (MilkoScan 7 RM, Eurofins Steins Laboratorium A/S, Vejen, Denmark). Milk yield expressed in ECM (3.140 MJ/kg) was calculated as ECM (kg) = milk yield × [(38.3 × fat + 24.2 × protein + 15.71 × lactose + 20.7)/3,140], with ECM and milk yield in kilograms; and fat, protein, and lactose monohydrate in grams per kilogram.

Four individual transparent respiration chambers based on open-circuit indirect calorimetry were used for measurement of gas exchange on d 5 to 7 in each period. The chambers were modified versions of Hellwing et al. (2012), and were operated as described by Thorsteinsson et al. (2023). The cows were assigned to the same specific respiration chamber throughout the experiment. Before, during, and after the experiment, recovery tests (n = 17 for CO_2_ and n = 18 for CH_4_) were performed by infusing a known amount of pure CO_2_ and CH_4_ into the chambers and comparing it with the amount of gas measured by the system. Across chambers, average recovery values ± SD were 98.6 ± 1.66% for CO_2_ and 99.8 ± 1.84% for CH_4_. Recovery tests were used to correct the measured gas concentrations. The average of CH_4_ and CO_2_ recoveries was used to correct O_2_ and H_2_. Gas exchange was measured as flows at standard temperature and pressure (STP; 0°C/273.15 K and 101.325 kPa) and subsequently converted into grams per day as described by Thorsteinsson et al. (2023). The respiratory coefficient was calculated as the ratio between CO_2_ produced and O_2_ consumed (L/L), and gas yield and intensity were calculated based on the DMI and ECM yield, respectively, during each chamber period.

All statistical analyses were conducted in R v.4.3.2 (R Core Team, 2024). For all parameters displayed in tables, the observations were averaged within cows and periods, resulting in a dataset with 16 observations; however, due to an unexpected lower growth rate of *B. hamifera* in the final stage of the experiment and hence lack of biomass delivered in the fourth batch, the cow initially allocated to the MED diet in period 4 was fed CON instead. The effect of the seaweed inclusion on the various animal responses was analyzed with the following linear mixed model fitted:

*Y_dpc_* = *μ* + *α_d_* + *γ_p_* + *A_c_* + *Ε_dpc_*,

where *Y_dpc_* is the dependent response variable, *μ* is the overall mean, *α* is the fixed effect of diet (*d* = CON, LO, MED, or HI), *γ* is the fixed effect of period (*p* = 1 to 4), *A* is the random effect of cow (*c* = 1 to 4), and *Ε_dpc_* is the random residual error assumed to be independent with constant variance and normally distributed.

To obtain diurnal patterns of CH_4_ and H_2_, hourly emissions of the gases were averaged over the 3-d measurement period within cow and period, resulting in 24 observations per cow per period. The data were analyzed with the following model:

*Y_dhpc_* = *μ* + *α_d_* + *τ_h_* + *α_t_* × *τ_h_* + *γ_p_* + *A_c_* + *Ε_dhpc_*,

where *Y_dhpc_* is the dependent response variable, *μ* is the overall mean, *α* is fixed effect of treatment (*d* = CON, LO, MED, or HI), *τ* is the fixed effect of hour (*h* = 0 to 23), *α_t_* × *τ_h_* is the interaction, *γ* is the fixed effect of period (*p* = 1 to 4), *A* is the random effect of cow (*c* = 1 to 4), and *Ε_dhpc_* is the random residual error assumed to be independent with constant variance and normally distributed. Data were analyzed as repeated measurements using a first-order autoregressive covariance structure with heterogeneous variance (AR1).

All data were evaluated for normality and homogeneity of the variance during the statistical analysis. Data are presented in tables as estimated marginal means and SEM. Differences between estimated marginal means were evaluated using Tukey's method for comparison. Statistical significance was declared when *P* ≤ 0.05 and statistical tendencies were declared when 0.05 < *P* ≤ 0.10.

To the best of our knowledge, this is the first in vivo study evaluating the enteric CH_4_-mitigating potential of the red seaweed species *B. hamifera*. We are not aware of the existence of any large-scale commercial cultivation or harvest of *B. hamifera* anywhere in the world, and due to limited production capacity in a quite newly established Danish land-based facility, we were limited to a duration of 7 d of the individual experimental periods in the present pilot study. The CH_4_-mitigating potential of feed additives is often overestimated in vitro versus obtained reductions in vivo (Honan et al., 2022). Therefore, despite the short duration of experimental periods, this first in vivo experiment provides a far better foundation for the estimation of the antimethanogenic potential of *B. hamifera* than in vitro trials, which would have been the alternative with the seaweed supply limitations. Nonetheless, it is important to stress that only the short-term effects have been evaluated and will be discussed in the following sections, and that larger and longer-term in vivo trials should be undertaken.

Increasing inclusion levels of *B. hamifera* linearly decreased CH_4_ emissions with up to 13.3% reduction in daily CH_4_ production (g/d), 15.7% reduction in CH_4_ yield (g/kg DMI), and 16.4% reduction in CH_4_ intensity (g/kg ECM; Table 2). A similar dose-dependent response of *B. hamifera* on CH_4_ production has been reported in vitro by Mihaila et al. (2022) and Guinguina et al. (2023). From Figure 1, it can be observed that the lower daily CH_4_ production, yield, and intensity observed on the HI diet compared with CON was caused by a generally lower production throughout the day. In alignment with studies investigating *Asparagopsis* spp. as antimethanogenic feed additives (Roque et al., 2019; Stefenoni et al., 2021; Krizsan et al., 2023), reductions in CH_4_ emissions also resulted in increased H_2_ emissions in the current study. Hence, HI resulted in a 331% increase in daily H_2_ emission (g/d), 325% increase in H_2_ yield (g/kg DMI), and a 324% increase in H_2_ intensity (g/kg ECM; (Table 2).

**Table 2:** Dry matter intake and gas exchange of dairy cows fed increasing amounts of the red seaweed *Bonnemaisonia hamifera*

Item	Treatment^1^				SEM^2^	*P*-value	
	CON	LO	MED	HI		Treatment	Linear
DMI (kg/d)	23.1	23.5	23.4	23.7	1.72	0.63	0.30
Milk (kg)	28.2	29.1	29.6	29.1	4.72		0.22
ECM (kg/d)	27.1	28.1	28.6	27.8	4.23		0.22
Gas exchange (g/d)							
CO_2_	15,371	15,283	15,263	15,387	915	0.90	0.96
O_2_	10,274	10,270	10,267	10,326	569	0.96	0.72
CH_4_	384^a^	362^ab^	348^ab^	333^b^	34.7	0.04	<0.01
H_2_	1.79^b^	2.29^b^	4.24^ab^	7.71^a^	1.51	0.03	<0.01
Respiration coefficient^3^	1.09	1.08	1.08	1.08	0.0152	0.55	0.34
Gas yield (g/kg DMI)							
CO_2_	667	652	656	652	12.9	0.32	0.19
O_2_	447	439	442	438	14.0	0.94	0.77
CH_4_	16.6^a^	15.5^ab^	14.8^ab^	14.0^b^	0.930	0.01	<0.01
H_2_	0.0789^b^	0.0983^ab^	0.190^ab^	0.335^a^	0.0611	0.04	<0.01
Gas intensity (g/kg ECM)							
CO_2_	587	568	561	572	51.0	0.58	0.39
O_2_	393	382	377	385	35.2	0.74	0.49
CH_4_	14.6^a^	13.4^ab^	12.7^ab^	12.2^b^	1.38	0.02	<0.01
H_2_	0.0715^b^	0.0873^ab^	0.163^ab^	0.303^a^	0.0652	0.04	<0.01

a,b Values within the same line with different superscripts differ (*P* < 0.05).

1 CON = control diet; LO = 0.33% *B. hamifera*; MED = 0.66% *B. hamifera*; HI = 1% *B. hamifera* on a DM basis.

2 Largest SEM is reported.

3 Calculated as ratio between CO_2_:O_2_ (L/L).

**Figure 1:**
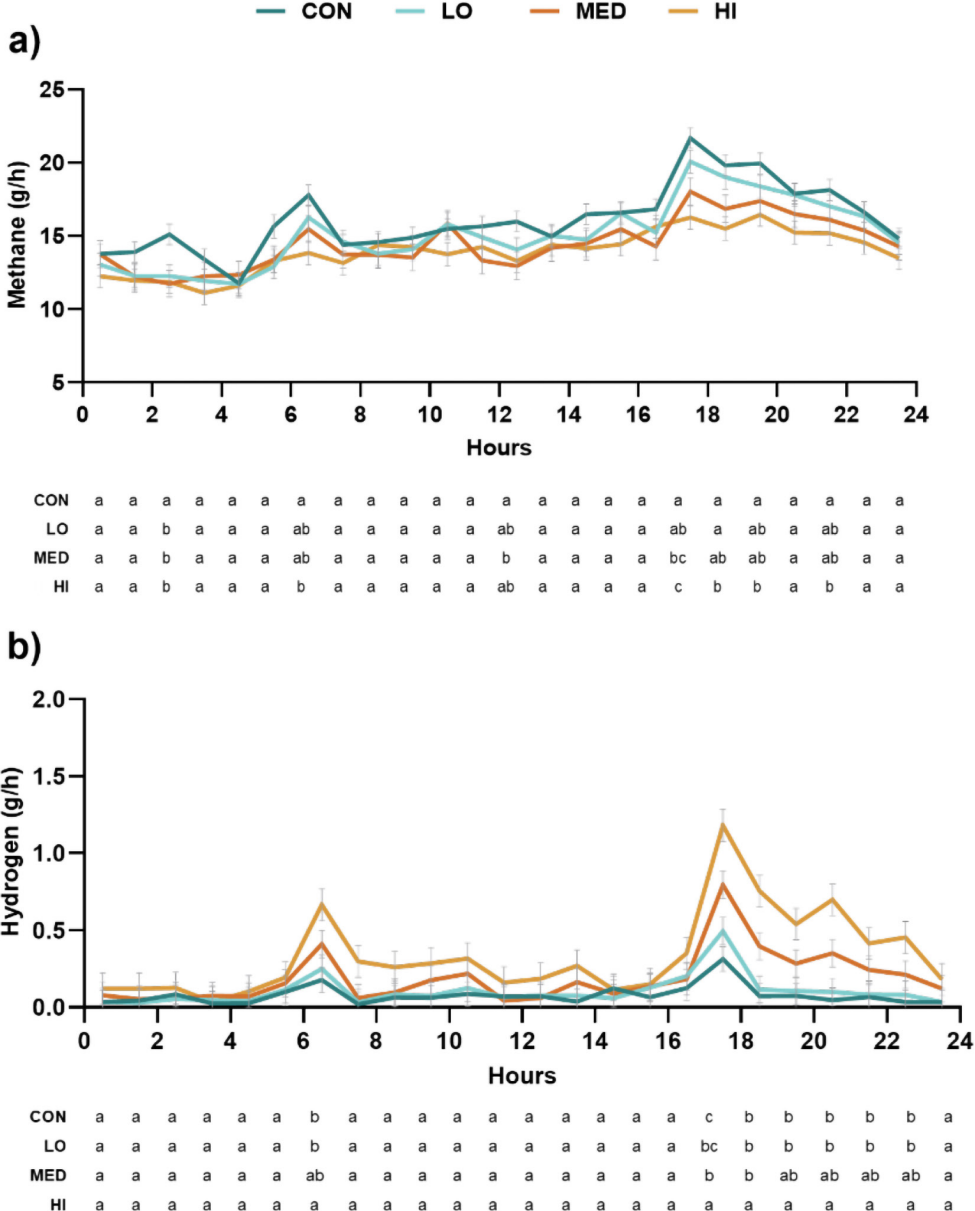
(a) Estimated hourly CH_4_ emission from dairy cows fed increasing amounts of *Bonnemaisonia hamifera* (mean ± SEM). (b) Estimated hourly H_2_ emission from dairy cows fed increasing amounts of *B. hamifera*. CON *=* control diet; LO = 0.33% *B. hamifera*; MED = 0.66% *B. hamifera*; HI = 1% *B. hamifera* on a DM basis. Each time point represents the average CH_4_ or H_2_ production from half an hour before to half an hour after that time (i.e., time point 0630 h represents the average gas production from 0600 to 0700 h). Different letters shown below the figures for a given time point indicate significantly different values between treatments at *P* < 0.05.

Studies with *Asparagopsis* spp. have demonstrated reductions in CH_4_ yield from dairy cows ranging from 43% to 54% at an inclusion level of up to 1% on an OM basis (Roque et al., 2019; Krizsan et al., 2023). In the current study, HI (1% on a DM basis) corresponds to approximately 0.9% OM inclusion rate of *B. hamifera*. Thus, *B. hamifera* appears to be a less potent seaweed compared with *Asparagopsis* spp. in terms of reducing enteric CH_4_ emission from dairy cows. The exact nature of the antimethanogenic compounds in *B. hamifera* is yet to be resolved; however, the concentration of bromoform in *B. hamifera* in the current study was low compared with reported levels in studies evaluating *Asparagopsis* spp. as CH_4_ inhibitor in dairy cows (Roque et al., 2019; Muizelaar et al., 2021). Hence, reported bromoform concentrations in *Asparagopsis* spp. across studies have been around 1.29 ± 0.04 mg/g DM (Roque et al., 2019; Muizelaar et al., 2021) in contrast to 0.101 ± 0.027 mg/g DM in *B. hamifera* biomass in the present study. This might explain the lower CH_4_-mitigating potential of *B. hamifera* compared with observed reductions in studies with *Asparagopsis* spp., but it should be noted that the difference between reduction potential is not proportional with bromoform concentration, indicating that other antimethanogenic compounds may be present in *B. hamifera.* Untargeted metabolomic analysis followed by in vitro fermentation studies should be undertaken to further identify and validate the antimethanogenic compounds in *B. hamifera.* Bromoform is categorized as a potential human carcinogen by the US Environmental Protection Agency (2000), and thus, as discussed by Wasson et al. (2022), concerns could be raised regarding the long-term impact of feeding *Asparagopsis* spp. on animal health as dairy cows persist within a herd for several years. Therefore, *B. hamifera* could potentially be a safer candidate for a seaweed-based CH_4_-mitigating feed additive in both in relation to animal and possibly also consumer health than *Asparagopsis* spp.

The inclusion of the red seaweed *Bonnemaisonia hamifera* in diets of dairy cows in this preliminary study resulted in dose-dependent reductions of daily CH_4_ production, CH_4_ yield, and CH_4_ intensity amounting to 13% to 16% reduction at the highest inclusion (1% in dietary DM) compared with the control diet. The concentration of the potentially harmful compound, bromoform, was remarkably lower in *B. hamifera* compared with previously reported concentrations in the antimethanogenic red seaweed, *Asparagopsis taxiformis.* Thus, it is highly relevant to undertake larger and longer-term in vivo trials to be able to fully investigate the potential of *B. hamifera* as a CH_4_ inhibitor in cattle.

## References

[bib1] Åkerlind M., Weisbjerg M.R., Eriksson T., Tøgersen R., Udén P., Òlafsson B.L., Harstad O.M., Volden H., Volden H. (2011). NorFor—The Nordic feed evaluation system. EAAP publication no. 130.

[bib2] Alvarez-Hess P.S., Jacobs J.L., Kinley R.D., Roque B.M., Neachtain A.S.O., Chandra S., Russo V.M., Williams S.R.O. (2024). Effects of a range of effective inclusion levels of *Asparagopsis**armata* steeped in oil on enteric methane emissions of dairy cows. Anim. Feed Sci. Technol..

[bib3] Enge S., Nylund G.M., Harder T., Pavia H. (2012). An exotic chemical weapon explains low herbivore damage in an invasive alga. Ecology.

[bib4] Guinguina A., Hayes M., Gröndahl F., Krizsan S.J. (2023). Potential of the red macroalga *Bonnemaisonia hamifera* in reducing methane emissions from ruminants. Animals (Basel).

[bib5] Hellwing A.L.F., Lund P., Weisbjerg M.R., Brask M., Hvelplund T. (2012). Technical note: Test of a low-cost and animal-friendly system for measuring methane emissions from dairy cows. J. Dairy Sci..

[bib6] Honan M., Feng X., Tricarico J.M., Kebreab E. (2022). Feed additives as a strategic approach to reduce enteric methane production in cattle: Modes of action, effectiveness and safety. Anim. Prod. Sci..

[bib7] Jia Y., Quack B., Kinley R.D., Pisso I., Tegtmeier S. (2022). Potential environmental impact of bromoform from *Asparagopsis* farming in Australia. Atmos. Chem. Phys..

[bib8] Kinley R.D., de Nys R., Vucko M.J., Machado L., Tomkins N.W. (2016). The red macroalgae *Asparagopsis taxiformis* is a potent natural antimethanogenic that reduces methane production during *in vitro* fermentation with rumen fluid. Anim. Prod. Sci..

[bib9] Krizsan S.J., Ramin M., Chagas J.C.C., Halmemies-Beauchet-Filleau A., Singh A., Schnürer A., Danielsson R. (2023). Effects on rumen microbiome and milk quality of dairy cows fed a grass silage-based diet supplemented with the macroalga *Asparagopsis taxiformis.*. Front. Anim. Sci..

[bib10] Lanigan G. (1972). Metabolism of pyrrolizidine alkaloids in the ovine rumen. IV. Effects of chloral hydrate and halogenated methanes on rumen methanogenesis and alkaloid metabolism in fistulated sheep. Aust. J. Agric. Res..

[bib11] Machado L., Magnusson M., Paul N.A., Kinley R., de Nys R., Tomkins N. (2016). Identification of bioactives from the red seaweed *Asparagopsis**taxiformis* that promote antimethanogenic activity in vitro. J. Appl. Phycol..

[bib12] Mihaila A.A., Glasson C.R.K., Lawton R., Muetzel S., Molano G., Magnusson M. (2022). New temperate seaweed targets for mitigation of ruminant methane emissions: An in vitro assessment. Appl. Phycol..

[bib13] Muizelaar W., Groot M., van Duinkerken G., Peters R., Dijkstra J. (2021). Safety and transfer study: Transfer of bromoform present in *Asparagopsis taxiformis* to milk and urine of lactating dairy cows. Foods.

[bib14] Nørskov N.P., Bruhn A., Cole A., Nielsen M.O. (2021). Targeted and untargeted metabolic profiling to discover bioactive compounds in seaweeds and hemp using gas and liquid chromatography-mass spectrometry. Metabolites.

[bib15] Nylund G., Cervin G., Persson F., Hermansson M., Steinberg P., Pavia H. (2008). Seaweed defence against bacteria: A poly-brominated 2-heptanone from the red alga *Bonnemaisonia hamifera* inhibits bacterial colonisation. Mar. Ecol. Prog. Ser..

[bib16] R Core Team (2024). https://www.r-project.org/.

[bib17] Roque B.M., Salwen J.K., Kinley R., Kebreab E. (2019). Inclusion of *Asparagopsis armata* in lactating dairy cows' diet reduces enteric methane emission by over 50 percent. J. Clean. Prod..

[bib18] Siuda J.F., VanBlaricom G.R., Shaw P.D., Johnson R.D., White R.H., Hager L.P., Rinehart K.L. (1975). 1-Iodo-3,3-dibromo-2-heptanone, 1,1,3,3-tetrabromo-2-heptanone, and related compounds from the red alga *Bonnemaisonia**hamifera*. J. Am. Chem. Soc..

[bib19] Stefenoni H.A., Räisänen S.E., Cueva S.F., Wasson D.E., Lage C.F.A., Melgar A., Fetter M.E., Smith P., Hennessy M., Vecchiarelli B., Bender J., Pitta D., Cantrell C.L., Yarish C., Hristov A.N. (2021). Effects of the macroalga *Asparagopsis taxiformis* and oregano leaves on methane emission, rumen fermentation, and lactational performance of dairy cows. J. Dairy Sci..

[bib20] Thorsteinsson M., Weisbjerg M.R., Lund P., Bruhn A., Hellwing A.L.F., Nielsen M.O. (2023). Effects of dietary inclusion of 3 Nordic brown macroalgae on enteric methane emission and productivity of dairy cows. J. Dairy Sci..

[bib21] US Environmental Protection Agency (2000). Bromoform; CASRN 75–25–2. https://www.epa.gov/sites/default/files/2016-09/documents/bromoform.pdf.

[bib22] Volden H. (2011).

[bib23] Wasson D.E., Yarish C., Hristov A.N. (2022). Enteric methane mitigation through *Asparagopsis taxiformis* supplementation and potential algal alternatives. Front. Anim. Sci..

